# Exploring the Impact of Workplace Satisfaction, Leadership, and Career Development on Employee Retention in Hospitals

**DOI:** 10.7759/cureus.81493

**Published:** 2025-03-31

**Authors:** Aliz Ildıko Bradacs, Florica Voita-Mekeres, Lucia Georgeta Daina, Gabriel Mekeres

**Affiliations:** 1 Department of Health Sciences, Doctoral School of Biomedical Sciences, University of Oradea, Oradea, ROU; 2 Department of Morphological Disciplines, University of Oradea, Oradea, ROU; 3 Department of Psycho-Neurosciences and Recovery, University of Oradea, Oradea, ROU; 4 Faculty of Medicine and Pharmacy, University of Oradea, Oradea, ROU

**Keywords:** employee retainment, healthcare, leadership, training opportunities, workplace satisfaction

## Abstract

Background and objectives

This study examines hospital employees’ perceptions of workplace satisfaction, communication, and professional development, providing insights into key factors affecting job satisfaction, retention, and the overall work environment.

Methods

A longitudinal survey design was employed to assess hospital employees’ perceptions of workplace satisfaction, communication, and professional development at Bihor County Emergency Clinical Hospital. Data were collected over a four-year period (2019-2022) to capture evolving trends in employee attitudes and experiences. The total sample size includes 3,732 participants, who were asked to complete a questionnaire, with data stratified by year and analyzed for statistical significance using p-values.

Results

The survey results reveal strengths in infection control awareness and compliance, with 95% of employees understanding their responsibilities in preventing healthcare-associated infections. Positive interpersonal communication and collaboration were also highlighted, with 90.9% of employees reporting good relationships with colleagues. However, the survey also identified areas for improvement, particularly in career advancement opportunities, with only 41.8% of respondents believing the hospital had a structured promotion policy.

Conclusions

These findings suggest that while the hospital excels in certain aspects, addressing gaps in career development, resource management, leadership responsiveness, and training opportunities is essential for enhancing employee satisfaction, reducing turnover, and improving patient care outcomes. Recommendations include establishing merit-based promotion systems, improving infrastructure, and expanding professional development programs.

## Introduction

Workplace satisfaction and professional development are critical factors in maintaining a highly motivated and efficient workforce, particularly in the demanding field of healthcare [1]. Hospitals rely on their employees’ well-being, engagement, and professional growth to ensure high-quality patient care and optimal organizational performance. Factors such as communication, access to resources, career development opportunities, and efficient infection control protocols significantly impact healthcare professionals’ job satisfaction and productivity [2,3]. When these elements are effectively managed, they contribute to a more positive work environment, reducing burnout and enhancing the quality of healthcare services [4].

Studies conducted in public hospitals have shown that a positive work environment, characterized by effective communication, strong leadership support, and access to necessary resources, contributes to higher job satisfaction and reduced burnout among hospital staff [5-8]. Organizational culture and management strategies play a crucial role in shaping employees’ experiences, with open communication and recognition of professional achievements being key motivators [9]. Additionally, workplace satisfaction is closely linked to patient outcomes, as engaged and satisfied healthcare workers tend to provide more efficient and compassionate care [10].

Professional development and career advancement opportunities are also critical components of employee satisfaction in the healthcare sector. Research indicates that hospitals that invest in continuous education, structured promotion policies, and skill development programs tend to have a more motivated workforce [11,12]. Employees who receive pathways for career growth and training are more likely to remain engaged and committed to their roles [13]. Moreover, the usage of digital infrastructure, such as intranet portals and online learning platforms, has been increasingly recognized as an essential tool for professional development, enabling staff to stay updated with medical advancements and institutional policies [14-16]. Rapid implementation of an integrated eHealth system in an Australian hospital showed mostly positive effects, including improvements in accountability and data utilization [17]. Digital health interventions have demonstrated potential for supporting health workforce development in low- and middle-income countries [18].

Despite the recognized importance of these factors, many hospitals continue to struggle with issues related to workplace stress [19,20], inadequate career growth opportunities [21-23], and communication barriers between employees and management [24,25]. Some studies indicate that rural hospital staff experience more work stress than their urban counterparts [26,27], while others suggest minimal differences [28,29]. Addressing these gaps is essential for fostering a supportive work environment that enhances both individual job satisfaction and overall hospital efficiency.

To better understand these workplace dynamics, a comprehensive survey was conducted among hospital staff, examining various dimensions of their professional experiences. The survey sought to assess workplace satisfaction, organizational communication, digital infrastructure, infection control awareness, career growth opportunities, and overall workplace safety. By analyzing employees’ perspectives on these factors, the study aims to identify strengths within the hospital’s work environment, while also pinpointing areas that require improvement. The results of this survey provide valuable insights into the hospital's internal functioning, offering a foundation for developing strategies that support employee well-being and institutional growth.

This article provides a comprehensive analysis of the survey results, outlining key trends, strengths, and challenges within the hospital setting. By addressing the issues identified, hospital administrators can implement strategic improvements that enhance employee satisfaction, promote career development, and strengthen communication between staff and leadership. Investing in these areas is essential for creating a supportive and dynamic work environment that empowers healthcare professionals to deliver the highest standard of patient care.

## Materials and methods

Study design and setting

A longitudinal survey design was employed to assess hospital employees’ perceptions of workplace satisfaction, communication, and professional development at Bihor County Emergency Clinical Hospital. Data were collected over a four-year period (2019-2022) to capture evolving trends in employee attitudes and experiences. In addition, patient satisfaction questionnaires - specifically, the 2019 version with revised questions aligned with accreditation standards - were analyzed by the County Clinical Emergency Hospital Oradea (CCEHO), Oradea, Romania. The CCEHO is a tertiary-level public hospital located in northwestern Romania, providing medical assistance to approximately 200,000 inhabitants of the Municipality of Oradea and emergency services to a territorial population of approximately 600,000 [30].

Participants

The study targeted hospital staff from various departments, with a total of 3,732 participants completing the survey. The respondents represented a diverse workforce in terms of age, gender, and education. The majority of participants were female (78.3%), with a mean age of 44.3 years (SD = 15.5). Educational backgrounds varied, ranging from elementary education to university degrees. Participation was entirely voluntary, and all responses were collected anonymously. Eligible participants included staff from any department or professional group, such as doctors, nurses, nursing assistants, laboratory technicians, patient transporters, registrars, and support staff, who were directly involved in the hospital’s daily operations. In addition, participants needed to have provided informed consent and voluntarily completed the survey questionnaire, with their responses containing the essential sections (demographics, workplace satisfaction, communication, and professional development) necessary for statistical analysis.

The exclusion criteria eliminated individuals who were not employed as permanent or regular staff, including external contractors, temporary workers, interns, or volunteers, as these individuals did not have a direct, ongoing role in hospital operations. Employees who declined participation or did not provide informed consent were also excluded, as were incomplete survey responses that lacked critical information required for analysis, such as missing key demographic or outcome data. Furthermore, any responses failing internal consistency or quality control checks were not included in the final analysis.

Data collection instrument

The questionnaire comprised 37 standardized questions developed under the monitoring obligations of patient satisfaction, as outlined in the Framework Agreement regarding the conditions for the provision of medical assistance in the Romanian healthcare system [31]. The instrument was organized into seven domains: demographic data; accessibility/admission; hotel conditions; quality of medical care; patient safety and rights; overall satisfaction; and observations/suggestions. A structured questionnaire was designed based on established instruments and tailored to the hospital setting. It gathered demographic information (age, gender, education, and year of participation) and addressed aspects of digital infrastructure and communication (the usefulness of the hospital’s intranet portal and real-time access to necessary data). In addition, the instrument evaluated infection control awareness by assessing respondents’ understanding of infection prevention responsibilities, familiarity with national surveillance methodologies, and awareness of epidemiological risks. Finally, the questionnaire examined the workplace environment and professional development by exploring interpersonal relationships, career advancement opportunities, workplace safety, cleanliness, arrangement, and participation in training programs. The survey incorporated yes/no items, Likert-scale ratings, and open-ended questions. Prior to full-scale administration, the questionnaire was pilot-tested to ensure clarity and reliability.

Procedure

The survey was administered annually from 2019 to 2022 using both online platforms via the hospital’s intranet portal and paper-based formats to maximize accessibility. Participants received clear instructions regarding the purpose of the study, the voluntary nature of participation, and the assurance of confidentiality. Completion of the survey required approximately 15 to 20 minutes. Responses were collected, coded, and securely stored for subsequent analysis. To measure satisfaction levels, Likert scales with three, four, or five response options were used, depending on the questionnaire version. Respondents indicated their level of agreement either by selecting from descriptive categories (e.g., unsatisfactory, good, and very good) or by rating on a numerical scale from 1 to 5 [32-34].

Data analysis

All statistical analyses were conducted using R version 4.1.2 (R Foundation for Statistical Computing, Vienna, Austria). Descriptive statistics - including means, standard deviations, frequencies, and percentages - were computed to characterize the sample and summarize responses to key survey items, utilizing packages such as dplyr (v1.0.7; RStudio, Vienna, Austria) for data manipulation. Group differences for categorical variables, such as professional role and survey year, were assessed using Chi-square tests, while one-way analysis of variance (ANOVA) was employed to compare continuous variables like age across subgroups. A p-value less than 0.05 was considered statistically significant.

Internal consistency of the survey instrument was evaluated using Cronbach’s alpha (computed with the psych package v2.2.9), which yielded a value of 0.706, indicating acceptable reliability. Suitability for factor analysis was confirmed by the Kaiser-Meyer-Olkin (KMO) measure of sampling adequacy and Bartlett’s test of sphericity. Subsequently, principal component analysis (PCA) with varimax rotation was conducted to identify latent dimensions within the survey data, with decisions to retain components based on eigenvalues greater than 1, inspection of the scree plot, and the theoretical coherence of the extracted factors. Inter-component correlations were examined to verify the independence of the factors. This comprehensive analytical approach provided a robust framework for interpreting key domains influencing workplace satisfaction and organizational functioning.

Ethical considerations

The study was conducted in accordance with the Declaration of Helsinki and approved by the Institutional Review Board (or Ethics Committee) of County Clinical Emergency Hospital Oradea (no. 9406/08.04.2021). All patients agreed to participate in this study.

## Results

The study population consisted of 3,732 respondents, with a mean age of 44.3 years (SD = 15.5). The sample was predominantly female (2,924, or 78.3%), with increasing participation observed over the survey years - from 836 (22.4%) in 2019 to 1,346 (36.1%) in 2022. Educational attainment varied, with 1,442 (38.6%) holding a university degree, 1,332 (35.7%) completing high school, and nearly 776 (21%) not disclosing their educational background. Occupational distribution was diverse: nurses (1,589, or 42.6%) and doctors (693, or 18.6%) represented the largest groups, while smaller proportions were observed among laboratory technicians, patient transporters, and registrars. Departmental affiliations spanned both clinical and administrative areas.

Interpersonal relationships were highly rated, with over 90% of respondents (n = 3,392, or 90.9%) reporting positive interactions with colleagues. A similar proportion indicated a clear understanding of performance expectations, although less than half (n = 1,559, or 41.8%) perceived the existence of a structured employee promotion policy. Regarding professional development, approximately half of the respondents (n = 1,884, or 50.5%) found their career growth within the hospital satisfactory, albeit with notable variability among professional groups. Workplace safety and infrastructure received favorable ratings; a majority felt secure regarding material equipment (n = 2,866, or 76.8%), while workplace cleanliness (n = 2,370, or 63.5%) and arrangement (n = 2,017, or 54.0%) were generally rated as good. In terms of training, most participants rated professional training positively (n = 1,906, or 51.1%) and believed that their skills were well utilized (n = 1,729, or 46.3%). Additionally, high levels of awareness of workplace risks (n = 3,295, or 88.3%) and managerial responsiveness (n = 2,882, or 77.2%) were observed, although perceptions regarding the adequacy of promotion policies and professional development opportunities remained less favorable (Table 1).

**Table 1:** Survey Results

Category	Count (%)
Usefulness of intranet portal	
Yes	2789 (74.7%)
No	324 (8.7%)
No response	619 (16.6%)
Real-time access to necessary data	
Yes	3095 (82.9%)
No	365 (9.8%)
No response	272 (7.3%)
Awareness of infection prevention responsibilities	
Yes	3547 (95.0%)
No	72 (1.9%)
No response	113 (3.0%)
Knowledge of national surveillance methodologies	
Yes	3286 (88.0%)
No	213 (5.7%)
No response	233 (6.2%)
Awareness of epidemiological risks	
Yes	3352 (89.8%)
No	208 (5.6%)
No response	172 (4.6%)
Motivation	
Yes	2687 (72.0%)
No	382 (10.2%)
No response	663 (17.8%)
Communication with hierarchical superiors	
Yes	3277 (87.8%)
No	173 (4.6%)
No response	282 (7.6%)
Effective communication with hospital management	
Yes	2533 (67.9%)
No	524 (14.0%)
No response	675 (18.1%)
Good relationship with colleagues	
Yes	3392 (90.9%)
No	116 (3.1%)
No response	224 (6.0%)
Understanding of expectations from superiors	
Yes	3327 (89.1%)
No	153 (4.1%)
No response	252 (6.8%)
Hospital employee promotion policy	
Yes	1559 (41.8%)
No	1001 (26.8%)
No response	1172 (31.4%)
Professional development within the hospital	
Unsatisfactory	258 (6.9%)
Satisfactory	1884 (50.5%)
Advantageous	1051 (28.2%)
No response	539 (14.4%)
Safety of material equipment	
Yes	2866 (76.8%)
No	404 (10.8%)
No response	462 (12.4%)
Workplace cleanliness	
Unsatisfactory	108 (2.9%)
Satisfactory	1160 (31.1%)
Good	2370 (63.5%)
No response	94 (2.5%)
Workplace arrangement	
Unsatisfactory	276 (7.4%)
Satisfactory	1262 (33.8%)
Good	2017 (54.0%)
No response	177 (4.7%)
Satisfaction with professional training	
Did not participate	220 (5.9%)
Unsatisfactory	143 (3.8%)
Satisfactory	1248 (33.4%)
Good	1906 (51.1%)
No response	215 (5.8%)
Utilization of skills and competencies	
Low	114 (3.1%)
Medium	1258 (33.7%)
High	1729 (46.3%)
No response	631 (16.9%)
Awareness of workplace risks	
Yes	3295 (88.3%)
No	208 (5.6%)
No response	229 (6.1%)
Consideration of improvement suggestions	
Yes	2882 (77.2%)
No	250 (6.7%)
No response	600 (16.1%)
Opinion on the questionnaire	
Not good	170 (4.6%)
Good	2308 (61.8%)
Very good	544 (14.6%)
No response	710 (19.0%)

In our sample of 3,732 hospital employees, the mean age was 44.3 years, with a standard deviation of 15.5, and no significant differences in age across professional groups. For example, doctors had a mean age of 44.0 years, nurses 43.5 years, nursing assistants 45.2 years, patient transporters 45.3 years, registrars 43.6 years, support staff 45.3 years, and laboratory staff 46.5 years, all exhibiting similar age profiles.

In contrast, response patterns by survey year varied significantly (p < 0.001). Notably, all 42 laboratory staff responded in 2022. Nurses’ participation increased from 350 (22.0%) in 2019 to 593 (37.3%) in 2022. Similar trends were evident among nursing assistants, whose participation changed from 159 (34.1%) in 2019 to 34 (7.3%) in 2022, patient transporters from 37 (29.6%) in 2019 to 25 (20.0%) in 2022, registrars from 37 (35.9%) in 2019 to 9 (8.7%) in 2022, and support staff from 98 (13.7%) in 2019 to 441 (61.8%) in 2022.

Educational attainment also differed among groups (p < 0.001). A large majority of doctors were university-educated, with 593 (85.6%) holding a degree, whereas many nursing assistants (232, or 49.8%) and patient transporters (70, or 56.0%) reported high school as their highest level of education. Overall, 1,442 (38.6%) of respondents held a university degree, 1,332 (35.7%) completed high school, 776 (20.8%) did not disclose their education, and 182 (4.9%) had only elementary education (Table 2).

**Table 2:** Demographic Characteristics of Hospital Employees by Professional Group For age and year comparisons, F-values from a Linear Model ANOVA are reported; Pearson’s Chi-squared test (χ²) was used. ANOVA, analysis of variance

Variable	Doctor (N = 693)	Laborator (N = 42)	Nurse (N = 1589)	Nursing Assistant (N = 466)	Patient Transporter (N = 125)	Registrar (N = 103)	Support Staff (N = 714)	Total (N = 3732)	Test Statistic	p-value
Age									F = 1.71	0.2221
Mean (SD)	44.0 (30.0)	46.5 (6.3)	43.5 (9.3)	45.2 (10.2)	45.3 (9.7)	43.6 (11.1)	45.3 (11.2)	44.3 (15.5)		
Year									χ² = 535.2 (df = 18)	<0.001
2019	155 (22.4%)	0 (0.0%)	350 (22.0%)	159 (34.1%)	37 (29.6%)	37 (35.9%)	98 (13.7%)	836 (22.4%)		
2020	119 (17.2%)	0 (0.0%)	249 (15.7%)	109 (23.4%)	12 (9.6%)	19 (18.4%)	87 (12.2%)	595 (15.9%)		
2021	217 (31.3%)	0 (0.0%)	397 (25.0%)	164 (35.2%)	51 (40.8%)	38 (36.9%)	88 (12.3%)	955 (25.6%)		
2022	202 (29.1%)	42 (100.0%)	593 (37.3%)	34 (7.3%)	25 (20.0%)	9 (8.7%)	441 (61.8%)	1346 (36.1%)		
Studies									χ² = 1157	<0.001
Elementary	3 (0.4%)	1 (2.4%)	33 (2.1%)	80 (17.2%)	9 (7.2%)	2 (1.9%)	54 (7.6%)	182 (4.9%)		
High-school	2 (0.3%)	17 (40.5%)	718 (45.2%)	232 (49.8%)	70 (56.0%)	41 (39.8%)	252 (35.7%)	1332 (35.7%)		
No response	95 (13.7%)	7 (16.7%)	337 (21.2%)	130 (27.9%)	37 (29.6%)	18 (17.5%)	152 (21.3%)	776 (20.8%)		
University	593 (85.6%)	17 (40.5%)	501 (31.5%)	24 (5.2%)	9 (7.2%)	42 (40.8%)	256 (35.9%)	1442 (38.6%)		

Table 3 shows that digital infrastructure items - such as the intranet portal (with 74.7% overall affirmative responses) and real-time data access (82.9% overall) - differed significantly across professional groups (p < 0.001). Nearly all respondents reported high awareness of infection prevention responsibilities and national surveillance methodologies, with group differences also reaching significance (p < 0.001). In addition, responses on risk awareness, motivation, and effective communication with hospital management varied significantly (p < 0.001), whereas communication with hierarchical superiors approached significance (p = 0.059). By contrast, interpersonal relationships and understanding of performance expectations did not differ significantly among groups (p = 0.092 and p = 0.121, respectively), nor did the perception of the employee promotion policy (p = 0.107), suggesting a level of fairness and consistency in these areas. However, significant differences were observed across groups in responses regarding professional development, safety of material equipment (p = 0.005), workplace cleanliness and arrangement, professional training, skill utilization, and consideration of improvement suggestions (with responses related to suggestions from superiors differing at p = 0.01). Moreover, the overall opinion on the questionnaire varied significantly (p < 0.001). These findings are important because they indicate that while employees share similar views on interpersonal relationships and performance expectations, disparities in other critical aspects of the work environment may necessitate targeted interventions to ensure equitable opportunities and resources across all professional groups.

**Table 3:** Distribution of Responses to the Employee Satisfaction Questionnaire Items by Professional Group Pearson’s Chi‑squared test was used to assess differences in the distribution of responses across professional groups.

Questionnaire Item	Doctor (N = 693)	Laborator (N = 42)	Nurse (N = 1589)	Nursing Assistant (N = 466)	Patient Transporter (N = 125)	Registrar (N = 103)	Support Staff (N = 714)	Total (N = 3732)	Test Statistic	p-value
Usefulness of intranet portal									Χ²(12) = 254.31	<0.0011
Yes	560.0 (80.8%)	36.0 (85.7%)	1310.0 (82.4%)	249.0 (53.4%)	70.0 (56.0%)	86.0 (83.5%)	478.0 (66.9%)	2789.0 (74.7%)		
No	44.0 (6.3%)	3.0 (7.1%)	70.0 (4.4%)	91.0 (19.5%)	25.0 (20.0%)	3.0 (2.9%)	88.0 (12.3%)	324.0 (8.7%)		
No response	89.0 (12.8%)	3.0 (7.1%)	209.0 (13.2%)	126.0 (27.0%)	30.0 (24.0%)	14.0 (13.6%)	148.0 (20.7%)	619.0 (16.6%)		
Real-time access to necessary data									Χ²(12) = 37.26	<0.0011
Yes	543.0 (78.4%)	38.0 (90.5%)	1340.0 (84.3%)	398.0 (85.4%)	102.0 (81.6%)	86.0 (83.5%)	588.0 (82.4%)	3095.0 (82.9%)		
No	103.0 (14.9%)	4.0 (9.5%)	133.0 (8.4%)	27.0 (5.8%)	13.0 (10.4%)	10.0 (9.7%)	75.0 (10.5%)	365.0 (9.8%)		
No response	47.0 (6.8%)	0.0 (0.0%)	116.0 (7.3%)	41.0 (8.8%)	10.0 (8.0%)	7.0 (6.8%)	51.0 (7.1%)	272.0 (7.3%)		
Awareness of infection prevention responsibilities									Χ²(12) = 126.42	<0.0011
Yes	657.0 (94.8%)	42.0 (100.0%)	1553.0 (97.7%)	455.0 (97.6%)	116.0 (92.8%)	87.0 (84.5%)	637.0 (89.2%)	3547.0 (95.0%)		
No	18.0 (2.6%)	0.0 (0.0%)	7.0 (0.4%)	0.0 (0.0%)	3.0 (2.4%)	6.0 (5.8%)	38.0 (5.3%)	72.0 (1.9%)		
No response	18.0 (2.6%)	0.0 (0.0%)	29.0 (1.8%)	11.0 (2.4%)	6.0 (4.8%)	10.0 (9.7%)	39.0 (5.5%)	113.0 (3.0%)		
Knowledge of national surveillance methodologies									Χ²(12) = 195.42	<0.0011
Yes	582.0 (84.0%)	39.0 (92.9%)	1491.0 (93.8%)	437.0 (93.8%)	100.0 (80.0%)	79.0 (76.7%)	558.0 (78.2%)	3286.0 (88.0%)		
No	67.0 (9.7%)	0.0 (0.0%)	31.0 (2.0%)	6.0 (1.3%)	9.0 (7.2%)	12.0 (11.7%)	88.0 (12.3%)	213.0 (5.7%)		
No response	44.0 (6.3%)	3.0 (7.1%)	67.0 (4.2%)	23.0 (4.9%)	16.0 (12.8%)	12.0 (11.7%)	68.0 (9.5%)	233.0 (6.2%)		
Do you consider that you are well-informed and aware of the pot									Χ²(12) = 116.17	<0.0011
Yes	602.0 (86.9%)	40.0 (95.2%)	1493.0 (94.0%)	436.0 (93.6%)	107.0 (85.6%)	86.0 (83.5%)	588.0 (82.4%)	3352.0 (89.8%)		
No	55.0 (7.9%)	0.0 (0.0%)	43.0 (2.7%)	8.0 (1.7%)	11.0 (8.8%)	9.0 (8.7%)	82.0 (11.5%)	208.0 (5.6%)		
No response	36.0 (5.2%)	2.0 (4.8%)	53.0 (3.3%)	22.0 (4.7%)	7.0 (5.6%)	8.0 (7.8%)	44.0 (6.2%)	172.0 (4.6%)		
Motivation									Χ²(12) = 50.43	<0.0011
Yes	551.0 (79.5%)	29.0 (69.0%)	1141.0 (71.8%)	315.0 (67.6%)	87.0 (69.6%)	60.0 (58.3%)	504.0 (70.6%)	2687.0 (72.0%)		
No	59.0 (8.5%)	4.0 (9.5%)	156.0 (9.8%)	53.0 (11.4%)	23.0 (18.4%)	11.0 (10.7%)	76.0 (10.6%)	382.0 (10.2%)		
No response	83.0 (12.0%)	9.0 (21.4%)	292.0 (18.4%)	98.0 (21.0%)	15.0 (12.0%)	32.0 (31.1%)	134.0 (18.8%)	663.0 (17.8%)		
Communication with hierarchical superiors									Χ²(12) = 20.48	0.0591
Yes	613.0 (88.5%)	34.0 (81.0%)	1390.0 (87.5%)	407.0 (87.3%)	104.0 (83.2%)	92.0 (89.3%)	637.0 (89.2%)	3277.0 (87.8%)		
No	32.0 (4.6%)	3.0 (7.1%)	62.0 (3.9%)	24.0 (5.2%)	13.0 (10.4%)	3.0 (2.9%)	36.0 (5.0%)	173.0 (4.6%)		
No response	48.0 (6.9%)	5.0 (11.9%)	137.0 (8.6%)	35.0 (7.5%)	8.0 (6.4%)	8.0 (7.8%)	41.0 (5.7%)	282.0 (7.6%)		
Effective communication with hospital management									Χ²(12) = 36.18	<0.0011
Yes	486.0 (70.1%)	27.0 (64.3%)	1012.0 (63.7%)	338.0 (72.5%)	87.0 (69.6%)	80.0 (77.7%)	503.0 (70.4%)	2533.0 (67.9%)		
No	101.0 (14.6%)	4.0 (9.5%)	240.0 (15.1%)	66.0 (14.2%)	17.0 (13.6%)	6.0 (5.8%)	90.0 (12.6%)	524.0 (14.0%)		
No response	106.0 (15.3%)	11.0 (26.2%)	337.0 (21.2%)	62.0 (13.3%)	21.0 (16.8%)	17.0 (16.5%)	121.0 (16.9%)	675.0 (18.1%)		
Good relationship with colleagues									Χ²(12) = 18.86	0.0921
Yes	617.0 (89.0%)	36.0 (85.7%)	1437.0 (90.4%)	437.0 (93.8%)	112.0 (89.6%)	95.0 (92.2%)	658.0 (92.2%)	3392.0 (90.9%)		
No	33.0 (4.8%)	1.0 (2.4%)	47.0 (3.0%)	7.0 (1.5%)	6.0 (4.8%)	2.0 (1.9%)	20.0 (2.8%)	116.0 (3.1%)		
No response	43.0 (6.2%)	5.0 (11.9%)	105.0 (6.6%)	22.0 (4.7%)	7.0 (5.6%)	6.0 (5.8%)	36.0 (5.0%)	224.0 (6.0%)		
Understanding of expectations from superiors									Χ²(12) = 17.83	0.1211
Yes	611.0 (88.2%)	40.0 (95.2%)	1427.0 (89.8%)	413.0 (88.6%)	109.0 (87.2%)	94.0 (91.3%)	633.0 (88.7%)	3327.0 (89.1%)		
No	30.0 (4.3%)	1.0 (2.4%)	57.0 (3.6%)	12.0 (2.6%)	9.0 (7.2%)	5.0 (4.9%)	39.0 (5.5%)	153.0 (4.1%)		
No response	52.0 (7.5%)	1.0 (2.4%)	105.0 (6.6%)	41.0 (8.8%)	7.0 (5.6%)	4.0 (3.9%)	42.0 (5.9%)	252.0 (6.8%)		
Hospital employee promotion policy									Χ²(12) = 18.30	0.1071
Yes	298.0 (43.0%)	16.0 (38.1%)	654.0 (41.2%)	176.0 (37.8%)	60.0 (48.0%)	42.0 (40.8%)	313.0 (43.8%)	1559.0 (41.8%)		
No	176.0 (25.4%)	8.0 (19.0%)	433.0 (27.2%)	133.0 (28.5%)	33.0 (26.4%)	19.0 (18.4%)	199.0 (27.9%)	1001.0 (26.8%)		
No response	219.0 (31.6%)	18.0 (42.9%)	502.0 (31.6%)	157.0 (33.7%)	32.0 (25.6%)	42.0 (40.8%)	202.0 (28.3%)	1172.0 (31.4%)		
Professional development within the hospital									Χ²(18) = 88.53	<0.0011
Unsatisfactory	70.0 (10.1%)	2.0 (4.8%)	76.0 (4.8%)	41.0 (8.8%)	4.0 (3.2%)	10.0 (9.7%)	55.0 (7.7%)	258.0 (6.9%)		
Satisfactory	327.0 (47.2%)	22.0 (52.4%)	864.0 (54.4%)	221.0 (47.4%)	57.0 (45.6%)	47.0 (45.6%)	346.0 (48.5%)	1884.0 (50.5%)		
Advantageous	232.0 (33.5%)	7.0 (16.7%)	440.0 (27.7%)	113.0 (24.2%)	39.0 (31.2%)	21.0 (20.4%)	199.0 (27.9%)	1051.0 (28.2%)		
No response	64.0 (9.2%)	11.0 (26.2%)	209.0 (13.2%)	91.0 (19.5%)	25.0 (20.0%)	25.0 (24.3%)	114.0 (16.0%)	539.0 (14.4%)		
Safety of material equipment									Χ²(12) = 28.30	0.0051
Yes	496.0 (71.6%)	29.0 (69.0%)	1256.0 (79.0%)	364.0 (78.1%)	101.0 (80.8%)	78.0 (75.7%)	542.0 (75.9%)	2866.0 (76.8%)		
No	103.0 (14.9%)	7.0 (16.7%)	153.0 (9.6%)	40.0 (8.6%)	7.0 (5.6%)	9.0 (8.7%)	85.0 (11.9%)	404.0 (10.8%)		
No response	94.0 (13.6%)	6.0 (14.3%)	180.0 (11.3%)	62.0 (13.3%)	17.0 (13.6%)	16.0 (15.5%)	87.0 (12.2%)	462.0 (12.4%)		
Workplace cleanliness									Χ²(18) = 134.62	<0.0011
Unsatisfactory	39.0 (5.6%)	7.0 (16.7%)	32.0 (2.0%)	0.0 (0.0%)	1.0 (0.8%)	10.0 (9.7%)	19.0 (2.7%)	108.0 (2.9%)		
Satisfactory	258.0 (37.2%)	5.0 (11.9%)	522.0 (32.9%)	103.0 (22.1%)	35.0 (28.0%)	35.0 (34.0%)	202.0 (28.3%)	1160.0 (31.1%)		
Good	380.0 (54.8%)	28.0 (66.7%)	994.0 (62.6%)	351.0 (75.3%)	87.0 (69.6%)	56.0 (54.4%)	474.0 (66.4%)	2370.0 (63.5%)		
No response	16.0 (2.3%)	2.0 (4.8%)	41.0 (2.6%)	12.0 (2.6%)	2.0 (1.6%)	2.0 (1.9%)	19.0 (2.7%)	94.0 (2.5%)		
Workplace arrangement									Χ²(18) = 77.66	<0.0011
Unsatisfactory	69.0 (10.0%)	9.0 (21.4%)	108.0 (6.8%)	22.0 (4.7%)	1.0 (0.8%)	15.0 (14.6%)	52.0 (7.3%)	276.0 (7.4%)		
Satisfactory	267.0 (38.5%)	12.0 (28.6%)	542.0 (34.1%)	124.0 (26.6%)	36.0 (28.8%)	40.0 (38.8%)	241.0 (33.8%)	1262.0 (33.8%)		
Good	322.0 (46.5%)	18.0 (42.9%)	859.0 (54.1%)	293.0 (62.9%)	82.0 (65.6%)	46.0 (44.7%)	397.0 (55.6%)	2017.0 (54.0%)		
No response	35.0 (5.1%)	3.0 (7.1%)	80.0 (5.0%)	27.0 (5.8%)	6.0 (4.8%)	2.0 (1.9%)	24.0 (3.4%)	177.0 (4.7%)		
Satisfaction with professional training									Χ²(24) = 316.31	<0.0011
Did not participate	43.0 (6.2%)	0.0 (0.0%)	30.0 (1.9%)	29.0 (6.2%)	4.0 (3.2%)	33.0 (32.0%)	81.0 (11.3%)	220.0 (5.9%)		
Unsatisfactory	53.0 (7.6%)	1.0 (2.4%)	47.0 (3.0%)	9.0 (1.9%)	5.0 (4.0%)	0.0 (0.0%)	28.0 (3.9%)	143.0 (3.8%)		
Satisfactory	234.0 (33.8%)	20.0 (47.6%)	591.0 (37.2%)	122.0 (26.2%)	51.0 (40.8%)	26.0 (25.2%)	204.0 (28.6%)	1248.0 (33.4%)		
Good	320.0 (46.2%)	21.0 (50.0%)	858.0 (54.0%)	271.0 (58.2%)	56.0 (44.8%)	30.0 (29.1%)	350.0 (49.0%)	1906.0 (51.1%)		
No response	43.0 (6.2%)	0.0 (0.0%)	63.0 (4.0%)	35.0 (7.5%)	9.0 (7.2%)	14.0 (13.6%)	51.0 (7.1%)	215.0 (5.8%)		
Utilization of skills and competencies									Χ²(18) = 52.10	<0.0011
Low	39.0 (5.6%)	0.0 (0.0%)	31.0 (2.0%)	19.0 (4.1%)	2.0 (1.6%)	7.0 (6.8%)	16.0 (2.2%)	114.0 (3.1%)		
Medium	227.0 (32.8%)	16.0 (38.1%)	512.0 (32.2%)	164.0 (35.2%)	52.0 (41.6%)	23.0 (22.3%)	264.0 (37.0%)	1258.0 (33.7%)		
High	322.0 (46.5%)	19.0 (45.2%)	778.0 (49.0%)	195.0 (41.8%)	52.0 (41.6%)	50.0 (48.5%)	313.0 (43.8%)	1729.0 (46.3%)		
No response	105.0 (15.2%)	7.0 (16.7%)	268.0 (16.9%)	88.0 (18.9%)	19.0 (15.2%)	23.0 (22.3%)	121.0 (16.9%)	631.0 (16.9%)		
Consideration of improvement suggestions									Χ²(12) = 36.22	<0.0011
Yes	597.0 (86.1%)	39.0 (92.9%)	1422.0 (89.5%)	432.0 (92.7%)	112.0 (89.6%)	92.0 (89.3%)	601.0 (84.2%)	3295.0 (88.3%)		
No	54.0 (7.8%)	2.0 (4.8%)	66.0 (4.2%)	15.0 (3.2%)	8.0 (6.4%)	6.0 (5.8%)	57.0 (8.0%)	208.0 (5.6%)		
No response	42.0 (6.1%)	1.0 (2.4%)	101.0 (6.4%)	19.0 (4.1%)	5.0 (4.0%)	5.0 (4.9%)	56.0 (7.8%)	229.0 (6.1%)		
Consideration of improvement suggestions from superior									Χ²(12) = 25.41	0.0131
Yes	528.0 (76.2%)	27.0 (64.3%)	1220.0 (76.8%)	366.0 (78.5%)	90.0 (72.0%)	87.0 (84.5%)	564.0 (79.0%)	2882.0 (77.2%)		
No	47.0 (6.8%)	5.0 (11.9%)	101.0 (6.4%)	31.0 (6.7%)	19.0 (15.2%)	4.0 (3.9%)	43.0 (6.0%)	250.0 (6.7%)		
No response	118.0 (17.0%)	10.0 (23.8%)	268.0 (16.9%)	69.0 (14.8%)	16.0 (12.8%)	12.0 (11.7%)	107.0 (15.0%)	600.0 (16.1%)		
Opinion on the questionnaire									Χ²(18) = 47.77	<0.0011
Not good	27.0 (3.9%)	3.0 (7.1%)	54.0 (3.4%)	22.0 (4.7%)	5.0 (4.0%)	3.0 (2.9%)	56.0 (7.8%)	170.0 (4.6%)		
Good	430.0 (62.0%)	22.0 (52.4%)	1043.0 (65.6%)	291.0 (62.4%)	71.0 (56.8%)	58.0 (56.3%)	393.0 (55.0%)	2308.0 (61.8%)		
Very good	103.0 (14.9%)	6.0 (14.3%)	216.0 (13.6%)	66.0 (14.2%)	25.0 (20.0%)	14.0 (13.6%)	114.0 (16.0%)	544.0 (14.6%)		
No response	133.0 (19.2%)	11.0 (26.2%)	276.0 (17.4%)	87.0 (18.7%)	24.0 (19.2%)	28.0 (27.2%)	151.0 (21.1%)	710.0 (19.0%)		

The analysis demonstrates significant differences among professional groups in most domains, particularly regarding digital infrastructure, infection control, and workplace conditions. Nurses and doctors generally reported more favorable perceptions of the intranet portal, real-time data access, and awareness of infection control measures, whereas nursing assistants, patient transporters, and support staff exhibited lower affirmative responses in several areas. Communication with hierarchical superiors and clarity of performance expectations were consistently high across groups, whereas effective communication with hospital management was rated less favorably overall. Additionally, while most respondents expressed satisfaction with workplace cleanliness and equipment safety, there remains notable variability in perceptions of professional development and recognition of skills. These findings underscore the heterogeneity in employee perceptions and provide a robust statistical basis for targeted quality improvement initiatives within the hospital.

A total of 3,732 participants contributed to the study. The KMO measure of sampling adequacy was 0.813, indicating that the sample was well-suited for factor analysis. Bartlett’s test of sphericity was significant (χ²(171) = 10,010, p < 0.001), supporting the assumption that the correlation matrix was factorable. PCA with varimax rotation identified four components with eigenvalues greater than 1, which together accounted for 42.6% of the total variance. Although 42.6% may seem modest, in the context of complex constructs such as organizational functioning and employee engagement, this level of variance explanation is generally considered acceptable. The scree plot confirmed that the sharp decline in eigenvalues after the fourth component justified retaining these four factors, and inter-component correlations were approximately zero, suggesting that the extracted factors were largely independent.

The first component, explaining 15.64% of the variance, was defined by items emphasizing communication and leadership, including communication with hierarchical superiors (loading = 0.687), effective communication with hospital management (0.624), and consideration of improvement suggestions from superiors (0.661). This domain appears to capture perceptions of managerial and supervisory interactions. The second component, which accounted for 11.07% of the variance, comprised items reflecting infection prevention awareness and knowledge, exemplified by knowledge of national surveillance methodologies (0.783) and awareness of infection prevention responsibilities (0.725). These loadings indicate that attitudes toward and familiarity with infection control measures clustered together as a distinct domain.

The third component explained 8.39% of the variance and included items pertaining to the physical work environment, such as workplace cleanliness (0.753) and workplace arrangement (0.767). Higher loadings in this factor suggest that participants’ perceptions of cleanliness, spatial organization, and related infrastructural aspects are closely interrelated. The fourth component, capturing 7.46% of the variance, was associated with professional development and skill usage. Items such as utilization of skills and competencies (0.694) and satisfaction with professional training (cross‐loading 0.436 on component 3 and 0.506 on component 4) clustered here, indicating that opportunities for growth and the application of competencies formed another coherent domain. Certain items, including the usefulness of the intranet portal (loading = 0.324), exhibited weaker loadings, implying that they did not align strongly with any single factor.

These four domains - managerial communication, infection prevention knowledge, workplace environment, and professional development - collectively explained a moderate proportion of the overall variance. Their near-zero inter-factor correlations suggest that they capture distinct dimensions of participants’ experiences. Although only 42.6% of the variance was explained, this level is generally considered acceptable in this research field, given the complexity of organizational and employee engagement constructs. The structure provides a meaningful framework for understanding key areas of organizational functioning and employee engagement, and the findings underscore the potential utility of evaluating each domain separately. Further refinements to item wording or domain coverage may strengthen the factor structure and clarify the role of items that demonstrated cross-loadings or relatively high uniqueness (Tables 4-7).

**Table 4:** Principal Component Analysis: Component Loadings and Uniqueness (Varimax Rotation) This table presents factor loadings (after varimax rotation) for each questionnaire item. Columns 1-4 show the loading values on the respective components, and the “Uniqueness” column indicates the proportion of variance unique to that item. A dash (“-”) denotes that no salient loading was observed for that component.

Item	Component 1	Component 2	Component 3	Component 4	Uniqueness
Usefulness of intranet portal	0.324	-	-	-	0.765
Real-time access to necessary data	0.425	-	-	-	0.733
Awareness of infection prevention responsibilities	-	0.725	-	-	0.46
Knowledge of national surveillance methodologies	-	0.783	-	-	0.366
Do you consider that you are well-informed and aware of the pot	-	0.743	-	-	0.403
Motivation	0.462	-	-	-	0.693
Communication with hierarchical superiors	0.687	-	-	-	0.52
Effective communication with hospital management	0.624	-	-	-	0.529
Good relationship with colleagues	0.522	-	-	-	0.709
Understanding of expectations from superiors	0.492	-	-	-	0.701
Hospital employee promotion policy	0.469	-	0.355	-	0.63
Professional development within the hospital	-	0.337	0.507	-	0.622
Safety of material equipment	0.433	-	-	-	0.741
Workplace cleanliness	-	0.753	-	-	0.428
Workplace arrangement	-	0.767	-	-	0.405
Satisfaction with professional training	-	0.436	0.506	-	0.543
Utilization of skills and competencies	-	0.694	0.512	-	-
Consideration of improvement suggestions	0.5	0.363	-	-	0.593
Consideration of improvement suggestions from superior	0.661	-	-	-	0.561

**Table 5:** Summary of Principal Components

Component	SS Loadings	% of Variance	Cumulative %
1	2.97	15.64	15.6
2	2.1	11.07	26.7
3	1.59	8.39	35.1
4	1.42	7.46	42.6

**Table 6:** KMO Measure of Sampling Adequacy (MSA) KMO: Kaiser-Meyer-Olkin

	MSA
Overall	0.813
Usefulness of intranet portal	0.856
Real-time access to necessary data	0.893
Awareness of infection prevention responsibilities	0.806
Knowledge of national surveillance methodologies	0.752
Do you consider that you are well-informed and aware of the pot	0.799
Motivation	0.87
Communication with hierarchical superiors	0.838
Effective communication with hospital management	0.834
Good relationship with colleagues	0.892
Understanding of expectations from superiors	0.889
Hospital employee promotion policy	0.83
Professional development within the hospital	0.726
Safety of material equipment	0.859
Workplace cleanliness	0.619
Workplace arrangement	0.622
Satisfaction with professional training	0.689
Utilization of skills and competencies	0.636
Consideration of improvement suggestions	0.86
Consideration of improvement suggestions from superior	0.854

**Table 7:** Initial Eigenvalues from Principal Component Analysis

Initial Eigenvalues			
1	3.602	18.96	19
2	1.844	9.71	28.7
3	1.535	8.08	36.7
4	1.104	5.81	42.6
5	1.036	5.45	48
6	0.951	5	53
7	0.908	4.78	57.8
8	0.849	4.47	62.3
9	0.792	4.17	66.4
10	0.787	4.14	70.6
11	0.739	3.89	74.5
12	0.712	3.75	78.2
13	0.692	3.64	81.9
14	0.657	3.46	85.3
15	0.612	3.22	88.5
16	0.578	3.04	91.6
17	0.566	2.98	94.6
18	0.554	2.92	97.5
19	0.481	2.53	100

## Discussion

The findings of this survey provide a comprehensive overview of hospital employees' perceptions regarding workplace satisfaction, communication, and professional development. While the results indicate strong compliance with infection control protocols and positive workplace relationships, they also reveal critical areas for improvement in career advancement opportunities, managerial responsiveness, and infrastructure.

A key strength identified in the survey is the high level of infection control awareness and compliance. With 95% of respondents understanding their responsibilities in preventing healthcare-associated infections and 88% being familiar with national surveillance methodologies, the results suggest that infection prevention training has been effective. Similar findings have been reported in studies conducted in European and North American hospitals, where strong infection control programs were associated with lower nosocomial infection rates and higher staff confidence in safety protocols [35-37]. These findings underline the importance of continuous education and training to sustain a high level of compliance and ensure that hospital staff remain well-informed about evolving infection control practices. Specific interventions that could further improve compliance include implementing e-learning modules that offer interactive, accessible training on infection control best practices. These modules could be integrated into mandatory training programs and regularly updated to reflect the latest guidelines. Additionally, scheduling regular refresher training sessions - both in-person and online - can help reinforce critical skills and knowledge. Simulation-based training, which provides hands-on practice in a controlled environment, may also be effective. Finally, incorporating periodic assessments and personalized feedback can identify areas for improvement and ensure that staff maintain high levels of compliance over time.

Another notable strength of the hospital environment is interpersonal communication and workplace relationships. The survey revealed that 90.9% of employees reported good collaboration with colleagues, and 87.8% found communication with hierarchical superiors to be effective. These results align with previous studies demonstrating that positive workplace relationships contribute to improved job satisfaction, reduced stress, and enhanced teamwork [38-40]. A study by Abdelhay et al. examined the impact of transformational leadership, career growth opportunities, work well-being, and work-life balance on nurse retention. The findings indicated that work-life balance and transformational leadership significantly influence nurse retention, highlighting the importance of supportive leadership and balanced work environments in retaining nursing staff [41].

Additionally, a study by van Kraaij et al. investigated the impact of differentiated nursing practices on the work environment and turnover intention in Dutch hospitals. The study found that enhancing work environments through tailored nursing roles can reduce turnover intention, suggesting that supportive work environments contribute to better employee retention [42]. These findings align with research by Gilmartin et al., which demonstrated that hospital leadership support plays a critical role in reducing burnout and fostering psychological safety among healthcare professionals. Their study highlighted that strong leadership engagement improves staff morale and contributes to a more positive work environment [43]. Additionally, Mohammed et al. found that physician leaders who practiced self-care and maintained professional fulfillment were more effective in their leadership roles, ultimately enhancing workplace culture and employee satisfaction [44].

Despite these strengths, the survey results highlight significant concerns regarding career advancement opportunities and professional development. Only 41.8% of employees believed that the hospital had a structured promotion policy, while 31.4% did not provide a response - potentially indicating uncertainty or dissatisfaction. This high non-response rate may reflect a lack of clarity regarding the promotion policy, a perception that current career advancement pathways are ambiguous, or even a reluctance to endorse what may be perceived as an inadequate system. These findings are in line with research showing that the absence of clear and equitable career progression is a major contributor to job dissatisfaction among healthcare workers [45]. Studies found that institutions with well-defined career pathways, mentorship programs, and regular performance evaluations reported higher job satisfaction and lower staff turnover [46,47]. These comparisons suggest that establishing transparent and merit-based promotion policies - including mentorship programs, structured career ladders, and leadership training - could enhance employee motivation and retention.

The survey also revealed challenges related to workplace infrastructure and resource availability. While 76.8% of employees felt they had access to necessary medical equipment and technology, a notable 10.8% expressed concerns. These concerns were often related to issues such as outdated technology, inadequate access to modern medical devices, and insufficient support from maintenance staff. Identifying these specific gaps can help guide targeted improvements to enhance workplace functionality and overall employee satisfaction. This issue has been widely documented in healthcare research. A study conducted in hospitals in South Africa found that resource shortages, particularly in rural areas, directly impacted healthcare workers' ability to perform their duties efficiently, leading to increased stress and job dissatisfaction [48]. Similarly, research in European hospitals indicated that hospitals with adequate equipment and well-maintained facilities reported significantly higher levels of employee satisfaction and patient safety outcomes [49]. Given these findings, hospital management should prioritize equitable resource distribution, regular facility maintenance, and infrastructure upgrades to ensure optimal working conditions.

Another concern highlighted in the survey is managerial responsiveness to employee concerns. While 77.2% of respondents felt their feedback was considered, 6.7% did not perceive hospital management as receptive. Studies indicate that leadership styles and employee engagement strategies significantly impact workplace satisfaction [50]. Research from UAE institutions found that hospitals that implemented a democratic leadership style, with structured feedback mechanisms such as regular staff consultations and participatory decision-making models, reported higher trust levels between employees and leadership [51]. Implementing structured leadership training, transparent feedback loops, and participatory governance models may, therefore, help improve managerial responsiveness in the hospital setting.

Lastly, training programs and skill utilization emerged as areas requiring attention. While 51.1% of respondents rated professional development courses as good, 5.9% reported not having participated in any training programs. Additionally, only 46.3% felt that their skills were well-utilized, suggesting potential underutilization of talent. These findings align with global research on professional development in healthcare settings. Studies have found that institutions investing in continuous education, cross-training, and leadership development programs experienced higher employee retention rates and better performance outcomes [52-54]. These comparisons suggest that the hospital could benefit from expanding training opportunities, ensuring equitable access to professional development programs, and creating pathways for employees to apply newly acquired skills in their daily work.

## Conclusions

The study reveals that hospital employees generally hold positive views regarding digital infrastructure, infection control, and interpersonal relationships, yet significant gaps persist in career development and managerial communication. Addressing these gaps may improve patient care, staff morale, and hospital efficiency. Targeted leadership development programs focused on effective communication, conflict resolution, and team building are recommended, along with structured feedback channels - such as anonymous surveys or regular town hall meetings - to gather candid employee input and guide continuous improvement. While most respondents demonstrated high awareness of infection prevention protocols and effective communication with peers and immediate supervisors, perceptions of leadership responsiveness and structured promotion policies were less favorable. Variations in responses among professional groups and distinct domains identified through factor analysis underscore the multifaceted nature of workplace satisfaction. Regular follow-up surveys and reassessments are essential to monitor progress and ensure that interventions lead to sustained improvements in the work environment.

## Appendices

**Table 8:** Employee Satisfaction Evaluation Questionnaire Credit: This table presents the Employee Satisfaction Evaluation Questionnaire used in the study. The questionnaire was adapted from the Framework Agreement regarding the conditions for the provision of medical assistance in the Romanian healthcare system [47]. Permission for its adaptation and use was obtained from the original publisher.

Employee Satisfaction Evaluation Questionnaire
«Location»
«Section/Department»
Dear Colleague,
In order to continuously improve the work environment and conditions, we are interested in your opinion regarding the level of professional satisfaction you experience at your workplace.
Please read the following statements carefully and respond by marking the answer that best reflects your opinion regarding your professional activity.
Mark an X in the box (□) corresponding to one of the answer options.
Please submit this questionnaire in the designated box located in the section where you work.
There is no need to sign it; this questionnaire is anonymous and confidential.
Your responses are important to us!
What is your PROFESSION?
□ Doctor □ Medical Assistant □ Social Worker □ Nurse □ Caregiver □ Medical Orderly □ Orderly + ADD □ Autopsy Technician □ Physiotherapist / Kinesiotherapist □ Pharmacist □ Biologist / Chemist □ Physicist □ Technician □ Other non-medical personnel □ TESA
Gender:
□ Female □ Male □ Prefer not to answer
Education:
□ Higher education □ Secondary education □ General education □ Prefer not to answer
Your age:
…… years □ Prefer not to answer
Do you consider the intranet portal to be useful for your work?
□ Yes □ No □ Prefer not to answer
Do you have real-time access to the data and information necessary to fulfill your responsibilities?
□ Yes □ No □ Prefer not to answer
Are you aware of your responsibilities regarding the prevention and control of infections associated with healthcare and communicable diseases?
□ Yes □ No □ Prefer not to answer
Are you familiar with the national methodologies for monitoring communicable diseases with nosocomial potential?
□ Yes □ No □ Prefer not to answer
Do you consider that you are informed and aware of the potential evolution and nosocomial risk of communicable diseases under epidemiological surveillance (e.g., Clostridium, influenza, etc.)?
□ Yes □ No □ Prefer not to answer
Are you motivated?
□ Yes □ No □ Prefer not to answer
Is there a communicative and collaborative relationship between you and your hierarchical superiors?
□ Yes □ No □ Prefer not to answer
Do you consider that there is an effective communication relationship between the hospital management and you?
□ Yes □ No □ Prefer not to answer
Do you consider that there is a good relationship between you, as an employee of the hospital, and your colleagues?
□ Yes □ No □ Prefer not to answer
As an employee, do you understand what outcomes your supervisors expect regarding your performance?
□ Yes □ No □ Prefer not to answer
Do you consider that there is a policy for promoting the hospital’s employees at the hospital level?
□ Yes □ No □ Prefer not to answer
What is your opinion regarding your professional development within the Hospital?
□ Unsatisfactory □ Satisfactory □ Advantageous □ Prefer not to answer
Do you feel secure regarding the availability of equipment, sanitary supplies, and apparatus necessary for the performance of your duties?
□ Yes □ No □ Prefer not to answer
How do you rate the cleanliness of your workplace?
□ Unsatisfactory □ Satisfactory □ Good □ Prefer not to answer
How do you rate the organization of your workplace?
□ Unsatisfactory □ Satisfactory □ Good □ Prefer not to answer
How satisfied are you with the quality and usefulness of the training sessions and professional development courses you have attended?
□ I have not attended □ Unsatisfactory □ Satisfactory □ Good □ Prefer not to answer
How do you rate the recognition of your skills and abilities?
□ Low □ Medium □ High □ Prefer not to answer
Do you consider that you are sufficiently informed about the risks associated with your workplace?
□ Yes □ No □ Prefer not to answer
Do you believe that your superior listens to you and considers your suggestions for improvement?
□ Yes □ No □ Prefer not to answer
What is your opinion about this questionnaire?
□ I do not have a good opinion □ Good □ Very good □ Prefer not to answer
COMMENTS OR SUGGESTIONS:
...............................................................................................................................................................................................................................................................
…………….………………..……………………………..………………………………………
